# Hospital Meetings, &c.

**Published:** 1899-10-07

**Authors:** 


### HOSPITAL MEETINGS, &C.

### LONDON SCHOOL OF TROPICAL MEDICINE.

A bright augury for the future of the London School of Tropical Medicine was the large gathering of scientists, students, and nurses at the new building—in the grounds of the Seamen's Hospital at the Albert Dock—on the occasion of what was termed on Monday afternoon (October 2nd) the "professional" inauguration of the institution. That is to say, the school at once starts its winter session, it being hoped that the formal opening, which is fixed for about May next, will be undertaken by a member of the Royal family.

Among those present at the opening ceremony, which proved to be a most pleasant gathering, were: Dr. R. Tanner-Hewlett, Dr. Guthrie Rankin, Dr. Andrew Duncan, and Dr. Oswald Baker, physicians of the hospital staff; Mr. James Cantlie, surgeon, with Professor W. J. Simpson and Dr. Westenra Sambon, of Naples, representing the school lecturers. Amongst others present were: Sir Frederick Young, Sir Charles Gage Brown, Surgeon-General Ross, Dr. Maxwell, and Mr. P. Michelli, the secretary.

It was unfortunate that Dr. Manson, who was to open the proceedings with an address, was unable to be present, but his able paper was read by Mr. Cantlie. The lecturer pointed out to the students that they were the nucleus of a body of men who would have an important influence upon the development of the empire, for with an adequate knowledge of tropical diseases would come the means of combatting them. Thus might the waste places of the earth become habitable and be developed. Many a medical man had gone out into his sphere in the tropics with bitter regret at his want of knowledge, which lack that school was intended to afford. The doctor emphasised the value of the proximity of the docks, as to the hospital would be brought all the cases providing studies of the various diseases of the tropical countries. Then the lecturer took a new line by pointing out that London was practically proof against the cholera epidemic by the careful protection of its water supply against infection; but, he added, it was not so immune against the plague, which was propagated by other means than man to man infection. Had this not been so its spread would long ago have been checked in India, where isolation was being properly carried out. They should look, then, to the hitherto unsuspected rat, which was unhampered by Government or police regulations in its wanderings from house to house. It behoved authorities to look to the destruction of rats, and thus add another link to the sanitary knowledge of the day.

After the address had been concluded, Sir Frederick Young proposed that the best thanks of the meeting be tendered to Mr. Chamberlain and to the Colonial Office for the contribution towards the new building, and for the assistance rendered in the establishment of the school. This having been carried, the proceedings terminated with thanks to Mr. P. A. Navine for presiding over the gathering.

